# Identification of a Novel Vasodilatory Octapeptide from the Skin Secretion of the African Hyperoliid Frog, *Kassina senegalensis*

**DOI:** 10.3390/molecules22071215

**Published:** 2017-07-19

**Authors:** Qiang Du, Hui Wang, Chengbang Ma, Yue Wu, Xinping Xi, Mei Zhou, Tianbao Chen, Chris Shaw, Lei Wang

**Affiliations:** 1 School of Pharmacy, China Medical University, Shenyang 110001, Liaoning, China; qdu@cmu.edu.cn; 2 Natural Drug Discovery Group, School of Pharmacy, Queen’s University, Belfast BT9 7BL, Northern Ireland, UK; c.ma@qub.ac.uk (C.M.); ywu16@qub.ac.uk (Y.W.); m.zhou@qub.ac.uk (M.Z.); t.chen@qub.ac.uk (T.C.); chris.shaw@qub.ac.uk (C.S.); l.wang@qub.ac.uk (L.W.)

**Keywords:** amphibian, skin secretion, peptide, smooth muscle

## Abstract

The defensive skin secretions of amphibians continue to be an excellent source of novel biologically-active peptides. Here we report the identification and pharmacological activity of a novel C-terminally amided myotropic octapeptide from the skin secretion of the African hyperoliid frog, *Kassina senegalensis*. The 8-amino acid peptide has the following primary structure: WMSLGWSL-amide and has a molecular mass of 978 Da. The primary structure and organisation of the biosynthetic precursor of WL-8 amide was successfully deduced from cloned skin secretion-derived cDNA. The open-reading frame encoded a single copy of WL-8, located at the C-terminus. Synthetic WL-8 amide was found to cause relaxation of rat tail artery smooth muscle with an EC_50_ of 25.98 nM. This peptide is unique in terms of its primary structure and is unlike any other peptide previously isolated from an amphibian source which has been archived in the NCBI database. WL-8 amide thus represents the prototype of a novel family of myotropic peptide from amphibian defensive skin secretions.

## 1. Introduction

The skin secretions of anurans (frogs and toads) have long been established as an excellent source of structurally-unique peptides which can be used as lead compounds in research into novel therapeutic agents [1,2]. These peptides have been honed through natural selection and physiological adaptations to aid the survival of the organism over millions of years [3].

A wide range of pharmacologically-active peptides are present in skin secretions. These peptides may exert some biological effect or even be highly toxic to would-be predators [3]. An example which demonstrates the physiological effects these peptides can have on predators has been reported in a study of the garter snake. The defensive secretions produced by *Xenopus* can cause oral dyskinesias in the snake, such that it cannot physically close its mouth [4]. This effect, if reproduced on similar predators in Nature, would certainly permit the frog to escape.

Many of the peptides isolated from amphibians exert their pharmacological functions by acting on specific targets within the body, for instance at G protein-linked receptors. This targeting ability of peptides makes them very interesting molecules in the search for potential drug candidates. The pharmacologically-active peptides found in the skin secretions of amphibians are often found to be analogues of the naturally-occurring regulatory or neuropeptides which carry out physiological functions in humans and in other vertebrates. Some are even identical to their endogenous mammalian counterparts such as thyrotropin-releasing hormone (TRH) [5] and the canonical bradykinin peptide, but most are not identical though they do have highly-conserved, biologically-active regions within their structures [6]. This latter group includes bombesin, which is an analogue of gastrin-releasing peptide in humans and also the caeruleins, which are similar to gastrin and cholecystokinin found in humans [7].

Frog skin secretion is a complex mixture of proteins and peptides which have a variety of pharmacological effects such as neurotoxicity and myotoxicity. Many proteins and polypeptides have been isolated from amphibian skin which exhibit a contractile effect on rat bladder smooth muscle. However, the effects of low molecular-weight peptides on such smooth muscles are not fully understood.

Here, the discovery of a prototype low molecular mass peptide from the skin secretion of the African hyperoliid frog, *Kassina senegalensis*, is reported. This peptide represents an entirely novel structural class of amphibian skin peptide and was found to display a unique relaxation of rat artery smooth muscle. The peptide was named, WL-8 amide (978 Da) in accordance with its chain length and first and last amino acid residues. We further describe the structure of its biosynthetic precursor identified through molecular cloning of its encoding cDNA from a skin secretion-derived cDNA library.

## 2. Results

**Figure 1:**
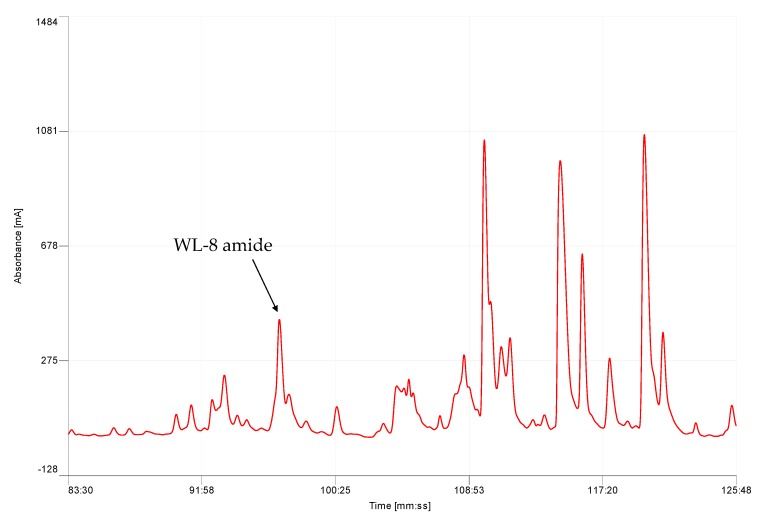
Region of reverse-phase high performance liquid chromatography (RP-HPLC) chromatogram of lyophilised *Kassina senegalensis* skin secretion. The retention time of the WL-8 peptide is 97 min (32.3% acetonitrile) and indicated by an arrow.

Each specimen of *K. senegalensis* yielded on average 30–35 mg dry weight of skin secretion following manual stimulation. Five mg of lyophilised skin secretion was subjected to reverse-phase high performance liquid chromatography (RP-HPLC) fractionation that produced a complex chromatogram (Figure 1).

**Figure 2:**
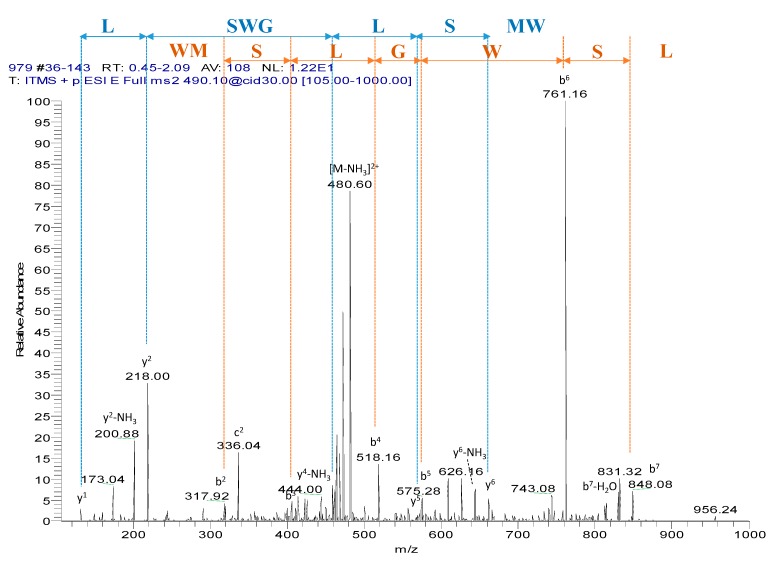
Annotated MS/MS fragmentation spectrum of natural WL-8 amide.

Chromatographic fractions were screened using the smooth muscle bioassay. The single peptide in fraction #97, which was eluted around 32% acetonitrile, exhibiting myotropic activity, was sequenced unambiguously by LC/MS/MS fragmentation (Figure 2). The amino acid sequence of WL-8 was determined as: Trp-Met-Ser-Leu-Gly-Trp-Ser-Leu-amide (WMSLGWSL).

**Figure 3:**
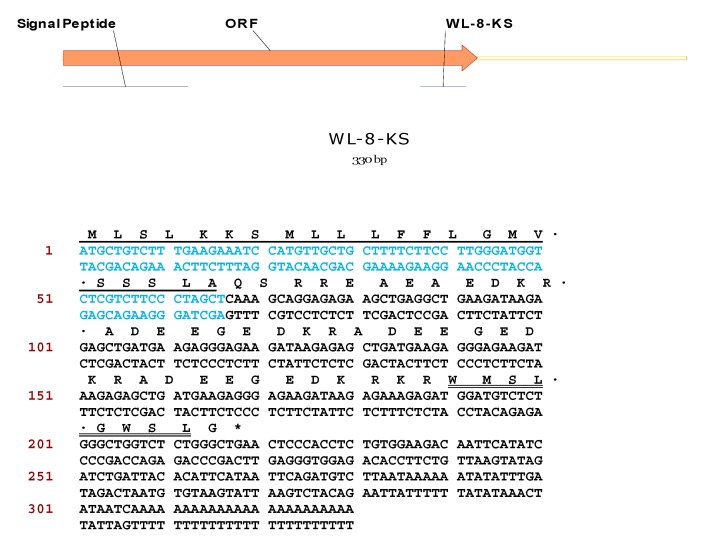
Nucleotide and translated open-reading frame amino acid sequence of full-length cDNA encoding a single copy of WL-8. The putative signal peptide (blue colour and single-underlined), the mature encoding sequence (double-underlined) and the stop codon (asterisk) are indicated.

The WL-8 amide-encoding precursor cDNA was successfully cloned from the skin secretion library using the RACE protocol described. The open-reading frame consisted of 76 amino acid residues (Figure 3). The mature peptide was flanked N-terminally by identical propeptide convertase processing sites probably involving a protease with Lys-Arg cleavage specificity. The last glycyl residue in the open-reading frame is appropriately positioned to act as an amide donor for generation of the C-terminal leucinamide residue present on the mature peptide. The nucleotide sequence of the WL-8 precursor has been deposited in the European Molecular Biology Laboratory (EMBL) Nucleotide Sequence Database under the accession code LT634112.

In order to produce a large quantity of replicate of the natural peptide, it was chemically-synthesised by solid phase Fmoc chemistry and the resultant peptide was lyophilized. The resulting product was of an apparent high degree of purity as determined by MALDI-TOF MS.

**Figure 4:**
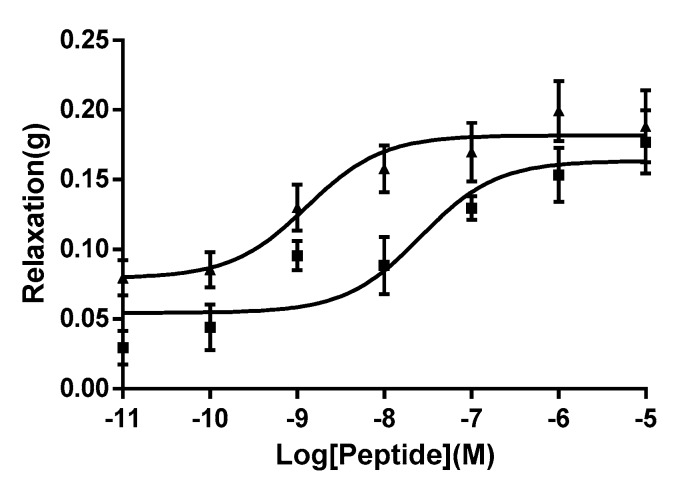
Dose–response curves of relaxation effects on phenylephrine stimulated rat tail artery smooth muscle preparation in the Bradykinin (▲) or WL-8 amide (■). Each point represents the mean and standard error. (4 repilicates, 3 individual experiments.) EC_50_ of Bradykinin: 1.29 nM; EC_50_ of WL-8 amide: 25.98 nM.

This synthetic replicate of WL-8 amide produced a dose-dependent relaxation of rat arterial smooth muscle though molar potencies were found to vary with preparations (Figure 4). A full dose response curve was constructed. Repeat experiments using the pure synthetic replicate in several different smooth muscle preparations showed that it was devoid of myorelaxant activity in these. In a further series of experiments, a dose-dependent relaxation was observed for WL-8 peptide in the range of 10^−11^–10^−5^ M with a maximal effective concentration of 10^−7^ M. Furthermore, the peptide was found to have an EC_50_ of 25.98 nM suggesting that this peptide is a very potent vasorelaxant.

## 3. Discussion

The skin secretions of amphibians continue to be an excellent source for the discovery of novel peptides [8,9]. The pharmacological effects of a novel amidated octapeptide, named WL-8, which was isolated from the skin secretions of the African running frog, *Kassina senegalensis*, were determined on smooth muscle preparations and the peptide was found to be a potent relaxant of vascular smooth muscle. An online protein/peptide database search revealed that no identical or even similar peptide sequence has yet been reported. Therefore, it was concluded that WL-8 is a novel biologically-active peptide. This peptide has two tryptophan (W) residues in its primary structure which is a rare finding in such a small peptide, additionally, it possesses two leucine residues and a single methionine. Thus 5 of the 8 amino acids that constitute this novel peptide are hydrophobic. Following a range of smooth muscle bioassays, the peptide was found to be a potent vasorelaxant (EC_50_ of 25.98 nM) in the rat tail artery as shown in Figure 4. 

As we used the pre-constricted rat tail artery smooth muscle by the stimulation of phenylephrine, the tissue could generate a low degree of spontaneous relaxant in the period time of equilibration. Therefore, we compared the vasorelaxant effect of WL-8 with bradykinin using the same tissue preparation and we can therefore confidently state that WL-8 peptide from *Kassina senegalensis* is a potent relaxant of arterial smooth muscle as it shown similar potency to bradykinin. This high degree of molar potency would suggest that it mediates its action via a specific molecular target, rather than through a generalised effect, for instance via membrane interaction. But the targeted receptors where WL-8 can specifically bind need to be further investigated.

Bioinformatic analysis of the structure of WL-8 amide found that it is unlike any other peptide previously isolated from an amphibian source. When the peptide is entered into the NCBI BLAST database, the closest matches from an animal source are the 38-mer cytokines, interferon gamma-1 and -2 isolated from rainbow trout (*Oncorhynchus mykiss*). The peptide exhibits a high degree of identity with the N-terminal regions of these fish cytokines. It may be that this peptide mimics their action in promoting inflammatory responses but this aspect may constitute a further series of studies. As this peptide is not structurally similar to anything previously isolated from an amphibian source its presence in the defensive secretions of *Kassina senegalensis* is most intriguing; it is unclear at this stage what its normal physiological action may be.

Bradykinin and structurally-related peptides are often significant components of amphibian defensive skin secretions and are found in representative species of the families, Ranidae, Hylidae and Bombinatoridae [6]. The actions of bradykinin are well known and diverse, such as vasodilation with subsequent induced hypotension, induction of pain and relaxation of a variety of smooth muscle types. However, the bradykinin and bradykinin-related peptides have not been found from the skin secretion of *Kassina* species yet (Uniport database, July 2017). In this study, the novel WL-8 peptide has similar myotropic effects to the bradykinin as it causes relaxation of smooth muscle; however, its structure is dissimilar to those of all the bradykinin like peptides previously discovered. Interestingly, we did not find any bradykinin or bradykinin related peptides from its skin secretion. It is speculated that different frogs evolved to produce specific-targeting peptides against the predators in their circumstances, of which WL-8 might induce similar or more potent effects than bradykinin in the bodies of the predators, and it eventually substituted for bradykinin in their defence strategy. 

Myotropic peptides are widely-distributed throughout the Animal Kingdom and play an important role in the tissues of animals [10]. Their effects are well known and diverse and include vasodilation with subsequent induced hypotension, induction of pain and relaxation of a variety of smooth muscle types. They exert their effects upon smooth muscle which is found in the gastrointestinal tract, the urinary tract and within the walls of blood vessels. There is therefore a potential to produce new therapeutic agents which will mediate some form of pharmacological effect at one of these sites, and so this peptide may have the potential to be used to treat diseases affecting at least one of these systems.

The African hyperoliid frog, *Kassina senegalensis*, has proven to be a valuable source of novel peptides which have the potential to be developed into new therapeutic agents [11,12]. Of the peptides isolated from this frog so far, some of those which show promise as potential therapeutic agents include the kassorins which possess antibiotic activity [12], and kassinin, a tachykinin possessing neurotransmitter and hormonal activity in higher vertebrates [13]. These discoveries, together with the novel peptide WL-8, indicate the potential this species has as a source for structurally-unique lead compounds or those that may reveal novel drug targets.

## 4. Materials and Methods

### 4.1. Specimen Biodata and Harvesting of Skin Secretion

Specimens of *K. senegalensis* (n = 12) were obtained from several sources. The frogs were adults (4–5 cm snout to vent length) and were kept for a period of 4 months prior to secretion harvesting. They were maintained in our purpose-designed amphibian facility at 18–25 °C under a 12 h/12 h light/dark cycle and fed multivitamin-loaded crickets three times per week. Defensive skin secretions were obtained from the dorsal skin surface by gentle transdermal electrical stimulation using the technique of Tyler et al. [14] under appropriate UK animal research licences. Briefly, the moistened skin was stimulated by platinum electrodes (6 V DC; 4 ms pulse-width; 50 Hz) for two periods of 20 s duration following which the secretion was washed from the skin using deionised water, snap-frozen in liquid nitrogen and lyophilised. Lyophilisate was stored at −20 °C prior to analysis. Sampling of skin secretion was performed by Mei Zhou under UK Animal (Scientific Procedures) Act 1986, project licence PPL 2694, issued by the Department of Health, Social Services and Public Safety, Northern Ireland. Procedures had been vetted by the IACUC of Queen’s University Belfast, and approved on 1 March 2011.

### 4.2. Fractionation of Skin Secretion by Reverse-Phase HPLC

A five milligram sample of lyophilised skin secretion from *K. senegalensis* was reconstituted in 0.05/99.5 (*v/v*) trifluoracetic acid (TFA)/water and clarified of particulates by centrifugation. The supernatant was subjected to fractionation by pumping directly onto an HPLC column (Jupiter C-5, 5 μm particle, 300 Å pore, 250 mm × 10 mm, Phenomenex, UK). The peptides were eluted using a gradient formed from 0.05/99.95 (*v/v*) TFA/water to 0.05/19.95/80.00 (*v/v/v*) TFA/water/acetonitrile in 240 min. A gradient reverse phase HPLC system was employed and fractions were collected automatically at 1 min intervals. Two 100 µL samples from each chromatographic fraction were removed, lyophilised and stored at −20 °C prior to analysis for smooth muscle and antimicrobial activities.

### 4.3. Rat Tail Artery Smooth Muscle Bioassay

Male Wistar rats (250–300 g) were euthanised by carbon dioxide asphyxiation followed by cervical dislocation under appropriate UK animal research licences. The rats were placed dorsal surface down and the abdomen was opened by means of an incision along the mid ventral line and subcutaneous fat was carefully dissected. The exposed urinary bladder was removed from each rat, emptied of urine and placed in ice-cold Kreb’s solution (118 mM NaCl, 4.7 mM KCl, 25 mM NaHCO_3_, 1.15 mM NaH_2_PO_4_, 2.5 mM CaCl_2_, 1.1 mM MgCl_2_ and 5.6 mM glucose), equilibrated with 95% O_2_, 5% CO_2_. A 2 mm width ring of rat tail artery was dissected under a dissection microscope and connected to two triangular hooks with a fine silk thread (0.2 mm diameter) with one end subsequently attached to a fixed pin and the other to a transducer in a 2 mL organ bath containing Kreb’s solution at 37 °C flowing at 2 mL/min with constant bubbling of 95% O_2_, 5% CO_2_. Muscle rings were pre-contracted with 10 µM phenylephrine and equilibrated for 1 h before experimental procedures were initiated. Following this, viable preparations were used to screen samples of reverse phase HPLC fractions of *K. senegalensis* skin secretion for myoactivity. Subsequently, once the novel active peptide had been identified, structurally characterised and chemically-synthesised, muscle strips were used to analyse dose–response relationships.

WL-8 amide solutions, ranging in concentration from 10^−11^ to 10^−5^ M, were made in Kreb’s solution and were used to construct a dose–response curve. These were added to the pre-constricted rat tail artery smooth muscle rings, using 10 µM phenylephrine, in increasing concentrations with 5 min equilibration periods between each dose. Each concentration of WL-8 amide solution was applied to a minimum of six muscle strips. Changes in tension of the artery smooth muscle rings were recorded and amplified through pressure transducers connected to a PowerLab System (AD Instruments Pty Ltd, Oxford, UK). Data were analysed to obtain the mean and standard error of responses by one-way ANOVA and dose–response curves were constructed using a “log(agonist) vs. response (three parameters), Least squares fit” algorithm through Prism 6 software package provided. 

### 4.4. Structural Characterisation Using MS/MS Fragmentation Sequencing 

The peptide in the reverse-phase HPLC fraction which was found to possess smooth muscle relaxing activity, was subjected to tandem MS/MS fragmentation sequencing using an LCQ-Fleet electrospray ion-trap mass spectrometer (Thermo Fisher Scientific, San Francisco, CA, USA). In detail, the sheath and auxiliary gas flow rates were set to 20 and 5 arbitrary units, respectively. The spray voltage was set to 4.50 kV while the capillary temperature was set to 275 °C. The fragmentation mode was collision-induced dissociation (CID) and normalised collision energy (NCE) was set as 35.

### 4.5. Molecular Cloning of WL-8 Amide Biosynthetic Precursor—Encoding cDNA

Five milligrams of lyophilised skin secretion were dissolved in 1 mL of cell lysis/mRNA protection buffer that was obtained from Invitrogen. Polyadenylated mRNA was isolated from this by using magnetic oligo-dT Dynabeads as described by the manufacturer (Invitrogen, Vilnius, Lithuania). The isolated mRNA was then subjected to 5′ and 3′-rapid amplification of cDNA ends (RACE) procedures to obtain full-length WL-8 amide precursor nucleic acid sequence data using a SMART-RACE kit (Clontech, Palo Alto, CA, USA) likewise as per manufacturer’s instructions. Briefly, the 3′-RACE reactions employed a nested universal (NUP) primer (supplied with the kit) and a degenerate sense primer (S: 5′-TGGATGWSIYTIGGITGG-3′) that was complementary to the N-terminal amino acid sequence, WMSLGW-, of the novel peptide, WL-8 amide. The 3′-RACE reactions were purified and cloned using a pGEM-T vector system (Promega Corporation, Southampton, UK) and sequenced using an ABI 3100 automated sequencer. The sequence data obtained from the 3′-RACE product was used to design a specific antisense primer (AS: 5′-GTCTTCCACAGAGGTGGGAGT-3′) to a defined conserved site within the 3′ non-translated region of the WL-8 amide encoding transcripts. 5′-RACE was carried out using these primers in conjunction with the NUP primer and resultant products were purified, cloned and sequenced.

### 4.6. Chemical Synthesis of WL-8 Amide

WL-8 amide was synthesised automatically using the solid-phase method (MBHA Rink Amide-resin, 0.6 mmol/g, Merck Millipore, Nottingham, UK) and 8 standard fluorenylmethoxycarbonyl (Fmoc) chemistry (double couplings with 8 equivalents of Fmoc-amino acid derivatives) using a PS3 automated solid phase peptide synthesiser (Protein Technologies, Tucson, AZ, USA). Couplings were performed using 2-(1*H*-benzotriazol-1-yl)-1,1,3,3-tetramethyluronium tetrafluoroborate (Calbiochem-Novabiochem AG, Läufelfingen, Switzerland).

Solid phase peptide synthesis begins with the covalent attachment (via its carboxyl group) of the C-terminal amino acid to an insoluble particle such as a polystyrene resin. The N-terminal end of the bound peptide undergoes coupling with carboxyl-activated amino-protected amino acids which results in the addition of residues to the expanding peptide, one amino acid at a time. Each step of this process includes a deprotection reaction and a coupling reaction. Upon completion of the peptide, it is chemically-cleaved from the resin. The peptide was purified by reverse-phase HPLC and its purity and molecular mass were confirmed using MALDI-TOF mass spectrometry.

## Abbreviations

The following abbreviations are used in this manuscript:

MDPI	Multidisciplinary Digital Publishing Institute
DOAJ	Directory of open access journals
TLA	Three letter acronym
cDNA	Complementary DNA
TRH	Thyrotropin-releasing hormone
RP-HPLC	Reverse-phase high performance liquid chromatography
BLAST	Basic Local Alignment Search Tool
NCBI	National Centre for Biotechnological Information
MALDI-TOF	Matrix-assisted laser desorption/ionisation, time-of-flight
MS	mass spectrometry
MS/MS	Tandem mass spectrometry
SEM	Standard error of the mean
TFA	Trifluoracetic acid
PS	Peptide synthesiser
LC/MS/MS	Liquid chromatography coupled tandem mass spectrometry
